# Not Just Vertigo?: A Case of Multi Territory Stroke with Atypical Cardiac Cause

**DOI:** 10.51894/001c.165990

**Published:** 2026-07-31

**Authors:** Alexandra Powers, Anirudhan Ramaseshan

**Affiliations:** 1 Henry Ford - Macomb

## 114

### Henry Ford - Macomb

Ischemic stroke is a leading cause of morbidity and mortality worldwide, particularly among older adults. Typical presentations include sudden onset hemiparesis, aphasia, facial droop, or sensory disturbances, reflecting the focal cerebral ischemia in specific vascular territories. Common causes are large artery atherosclerosis, cardioembolism, small vessel occlusion, and less commonly hypercoagulable states or rare embolic sources. However, atypical presentations can occur, complicating timely diagnosis and management. We present the case of a 73-year-old woman with a past medical history of vertigo and breast cancer, who developed a multi-territory ischemic stroke diagnosed on MRI. Notably, her initial presenting symptom was a severe bout of vertigo and transient right lower extremity weakness that led to a fall without classic neurological deficits. The delay in diagnosis caused by this atypical presentation is discussed. Comprehensive evaluation revealed multiple stroke infarcts in different vascular territories, including the posterior circulation, with no obvious arterial source, raising suspicion for a cardiac embolic origin complicated by a hypercoagulable state. Transesophageal echocardiography (TEE) with bubble study was performed to evaluate cardioembolic sources. Results showed 5 mm by 5 mm non-mobile vegetation is seen on the noncoronary cusp. An echogenic structure was identified predominantly involving the non-coronary cusp of the aortic valve and the ventricular side. There was evidence of mild prolapse, bowing, and mild eccentric aortic regurgitation. The etiology of this lesion is unclear, differential considerations included a thrombotic or infectious process, or a fibroelastoma. A cardiac MRI was subsequently planned to further assess the lesion. This report highlights the challenges in diagnosing atypical stroke presentations and emphasizes the importance of thorough multidisciplinary evaluation and management to prevent recurrence.

